# Precise Personalized Medicine in Gynecology Cancer and Infertility

**DOI:** 10.3389/fcell.2019.00382

**Published:** 2020-01-17

**Authors:** Pu-Yao Zhang, Yang Yu

**Affiliations:** ^1^Center for Reproductive Medicine, Department of Obstetrics and Gynecology, Peking University Third Hospital, Beijing, China; ^2^Beijing Key Laboratory of Reproductive Endocrinology and Assisted Reproductive Technology, Peking University Third Hospital, Beijing, China; ^3^Key Laboratory of Assisted Reproduction, Ministry of Education, Peking University Third Hospital, Beijing, China

**Keywords:** precision medicine, reproductive medicine, embryo, large-scale data, gynecologic cancer

## Abstract

Since the conception of precision medicine has been put forward in oncology, this idea has been popularized and applied in many specialties. Significant progress has been made toward personalizing the entire process, including diagnosis, treatment planning, and embryo identification, and combining large-scale genetic information data and knowledge discovery can offer better prospects in reproductive medicine. This work reviews the application of precision medicine and possibilities in reproductive medicine and gynecologic cancer diagnosis and treatment. The limitations and challenges of precision medicine in this area remain to be discussed.

## Introduction

Infertility is defined as the inability to achieve clinical pregnancy after 1 year of regular unprotected intercourse and can affect up to 14% of couples of reproductive age (Zegers-Hochschild et al., 2009). Reproductive medicine brings good news to many infertile couples. The first test-tube baby, Louise Brown, was born in 1978, and great progress has been made in improving the success rates and efficiency of fertility treatments over the past four decades. A number of new *in vitro* fertilization (IVF) practical regimens have been applied in different regions, and their viabilities still need to be evaluated. For example, embryo cryopreservation and subsequent thawed embryo transfer have replaced fresh embryo transfer (Roque et al., 2013), blastocyst-stage transfer has replaced cleavage-stage embryo transfer (Blake et al., 2007), single-embryo transfer (SET) has replaced double embryo transfer (DET) (Gelbaya et al., 2010), and preimplantation genetic screening (PGS) (Schoolcraft and Katz-Jaffe, 2013) and minimal stimulation protocols (Teramoto and Kato, 2007; Kato et al., 2012) have been applied.

**FIGURE 1:**
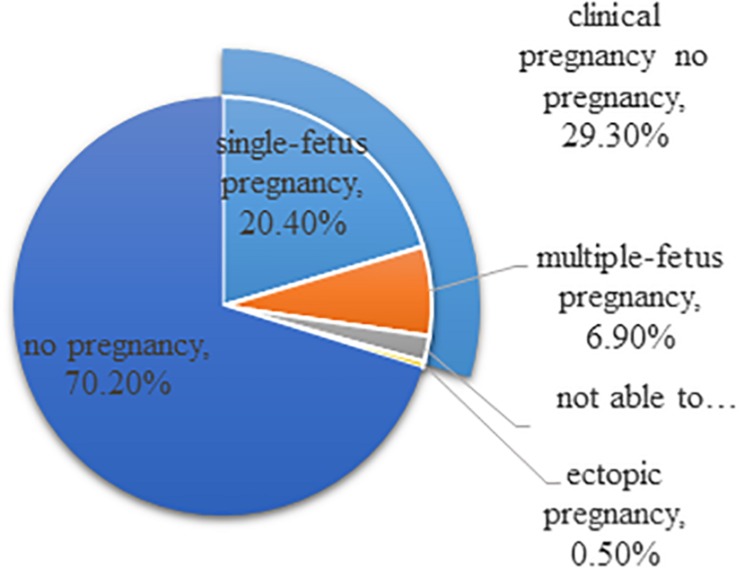
Outcomes of ART cycles using fresh non-donor eggs or embryos, 2015 (United States). The total does not equal 100% due to rounding.

Still, there is a lot of room for improvement of reproductive medicine, and it is required to be more cost-effective and efficient. In the United States, IVF treatments fail 70% of the time on a per cycle basis and cost $12,400 per treatment cycle on average (Figure 1; Centers for Disease Control and Prevention, 2011). It is estimated that approximately one in seven couples is struggling with infertility, but only a small fraction of these individuals could receive assisted reproductive technologies (ART) treatment (Beim et al., 2013). The number of patients who have received ART is increasing annually. An analysis focused on the development trend of assisted reproductive technology in Liaoning province from 2012 to 2016 shows that the number of fresh cycles and thawed cycles increased onefold. The success rate has been elevated, but it is still approximately 40%, which is consistent with the data provided from other regions. In summary, efficiency is a problem that urgently needs to be solved, and reproductive medicine should be more precise (Fang et al., 2018).

After experiencing evidence-based medicine (EBM), we have entered the era of precision medicine. EBM emphasizes the relationship between clinical research and clinicians, but the gap between clinicians and patients has not been filled. We have gradually realized that we need a new health care model that can adapt to various patients. The conception of this new health care model of precision medicine is beyond EBM. This conception started from oncology, which refers to selecting chemotherapeutic agents based on patients’ genetic profiles to maximize the efficiency and safety. Currently, the conception of precision medicine has been employed in many areas of medicine, such as psychiatry and cardiovascular disease. Genetic profiles have been identified that predict the necessary therapeutic dose for opioid analgesia and the amount of heart disease risk reduction a patient will gain from statins (Krawetz, 2017; Smith et al., 2017).

According to the definition promulgated by the National Institutes of Health (NIH), precision medicine refers to a thorough understanding of the consequences of unique features of individual patients, such as environmental exposures, lifestyle, and genetic profiles, and requires new individualized treatments and prevention methods. The goal of precision medicine is a better understanding and identification of patients who are at risk for particular diseases. It requires refinement of screening guidelines, to start early intervention and prevent unwanted outcomes and to urge potential patients to change their lifestyle and carefully consider overall life plans (Chang and Lee, 2018). In the realm of reproductive medicine, this conception refers to better information collection of health history, family history, lifestyle, and genotype, and customized treatment protocols are made based on this information. Instead of treating all patients with the same procedures, this type of personalized health model can increase the efficacy and efficiency of ART.

Precision medicine has already been widely accepted and implicated in reproductive medicine. However, reproductive medicine still faces severe challenges. The number of people facing fertility disorder is increasing gradually, and the reasons for fertility disorder are various. Reproductive medicine should be more precise in diagnosis, healthy embryo identification, treatment processes, and many other aspects. This review discusses the current application of precision medicine and the potential that exists for reproductive medicine.

## Precision Medicine Already Exists in Reproductive Medicine

The concept of precision medicine has been applied in reproductive medicine long before its popularization. The causes of infertility are various, and factors influencing the success rates of ART are complicated; hence, every step of reproductive medicine, such as the diagnosis of infertility causes and transfer of healthy embryos, needs to be precise.

### Preimplantation Genetic Diagnosis/PGS for Embryo Diagnosis

The ultimate goal of reproductive medicine is to achieve healthy offspring for infertile couples. Traditional ART overcomes the difficulties of infertility caused by organic diseases, such as oviduct obstruction, but that is far from enough for people with genetic disorders who want healthy offspring. Technologies that can screen out healthy embryos are required. Preimplantation genetic diagnosis/screening (PGD/PGS) offers the possibility.

Aneuploidies may occur in preimplantation embryos in any of the 24 chromosomes; for women receiving ART, this occurs in approximately 60% of abnormal embryos in women younger than 35 years, and it is up to 80% in women 41 years and older (Gutiérrez-Mateo et al., 2011; Yurttas Beim et al., 2017). Aneuploidy is considered as a significant factor in implantation failure and spontaneous miscarriage; thus, it might be the critical reason for IVF failure. It is necessary for a comprehensive aneuploidy screening before embryo implantation. Fluorescence *in situ* hybridization (FISH) can only check some specific chromosomes, usually chromosome 5, 9, or 12; thus, those missed aneuploidies might be the reason for embryo implantation failures. The emergence of new technologies brings new solutions for the problem of the disappointing clinical results of FISH, such as array comparative genomic hybridization (array-CGH), metaphase CGH, single-nucleotide polymorphism (SNP) microarrays (Fragouli et al., 2006), and quantitative polymerase chain reaction (Fiorentino et al., 2014). Array-CGH uses microarray technology to screen aneuploidy by detecting imbalances in 24 chromosomes (Gutiérrez-Mateo et al., 2011). The rapid development of next-generation sequencing (NGS) technologies has attracted interest in exploring their application in PGS. The clinical application of these comprehensive aneuploidy screening technologies has proven to be able to significantly improve consistency and predictive value for aneuploidy diagnosis and pregnancy outcomes (Treff et al., 2010; Fiorentino et al., 2011; Gutiérrez-Mateo et al., 2011). NGS might be more cost-effective and more precise than conventional technologies. The application of NGS in reproductive medicine improves the screening rate of healthy embryos and the success rate of pregnancy; thus, the whole process occurs in a more precise way (Table 1).

**TABLE 1:** Summary of the current methods used in PGD/PGS.

**Method**	**Application**	**Elevation**
FISH	Identifying chromosome translocations	Limited to common abnormalities involving chromosomes. High risk of error.
Multiplex qPCR	Chromosome copy number analysis	Reliable in determining aneuploidy. Not ideal for detecting structural chromosomal aberrations or uniparental disomy.
aCGH	Chromosome fragment duplication or deletion, chromosomal translocations	Can only detect translocated fragments that are >6 Mb.
SNP array	Blastocyst biopsy, embryo vitrification, chromosome screening, chromosomal translocations	Low pass, but strongly increases the reliability and stability of PGD.
NGS	Study an entire genome, identify almost all types of genetic variability	Quite costly but high pass, time-saving.
MALBAC	Aneuploidy and monogenic disorders of oocytes and polar bodies	Higher consistency and resolution. Not totally free of bias.
MARSALA	Aneuploidy, targeted mutation sites, SNPs	Avoids transmitting single-gene disorders to the next generation.

Many patients who seek assisted reproductive technology are with single-gene genetic diseases. There are approximately 7,000 known monogenic diseases, and the genes for more than half of these diseases have been identified. These patients need healthy embryos that do not carry disease-causing genes. The PGD precision has been limited by false-positive and false-negative single-nucleotide variations (SNVs). NGS not only applies to the screening of aneuploidies, but it can also be applied in the simultaneous evaluation of single-gene disorders, translocations, and abnormalities of the mitochondrial genome from the same biopsy without the need for multiple unique technological platforms. Mutated allele revealed by sequencing with aneuploidy and linkage analyses (MARSALA) combines NGS and single-cell whole-genome amplification methodologies to allow embryo diagnosis with single-molecule precision and benefits couples who desire to avoid transmitting their genetic diseases to their offspring (Yan et al., 2015). This technology allows the simultaneous direct observation of aneuploidy, targeted mutation sites, and their linked SNPs. MARSALA improved the precision of PGD prominently and streamlined the PGD/PGS procedure.

### Precision Medicine in Clinical Diagnosis and Treatment

A successful ART procedure requires not only healthy embryos but also precision in the whole process, including diagnosis and treatment. Fertility treatment centers are moving toward a personalized framework in assessing and treating patients that requires routinely incorporating genetic information and applying predicted models built on large-scale data in daily practice. A more detailed diagnosis and targeted treatment are needed to improve the success rates and make reproductive medicine more cost-effective.

#### Precise Identification of Endometrium Receptivity

The endometrium is a highly dynamic tissue. The embryo is unable to adhere to it through most of the menstrual cycle in humans, except during a short, self-limited period in which the endometrial tissue acquires a functional and transient receptive status that permits blastocyst adhesion (Psychoyos, 1986). Endometrium undergoes physiological changes in response to steroid hormones and genetic factors periodically to create a receptive status in a synchronized manner with the arrival of the implanting blastocyst during the window of implantation (WOI) between days 19 and 21. The endometrial epithelium undergoes specific structural, functional, and morphological changes to acquire a receptive phenotype (Murphy, 2004).

The correct identification of the appropriate WOI in a given patient using endometrial receptivity biomarkers can help to prevent reproductive failure resulting from poorly aligned timing with the WOI. A genomic tool named the endometrial receptivity array (ERA), based on a customized microarray, was developed, and a specially trained bioinformatic prediction computer algorithm was created to identify the WOI timing of the endometrium when it is specifically receptive to blastocyst adhesion. This genetic tool is designed to identify endometrial receptivity by comparing the genetic profile of a test sample with patients of LH+7 controls in natural cycles and patients on day 5 of P administration (P+5) after E2 priming in hormonal replacement therapy (HRT) cycles. This genetic tool is composed of a customized array containing 238 genes that are differentially expressed between these profiles above, which is coupled to a computational predictor that can diagnose the personalized endometrial WOI for a given patient without checking their endometrial histology (Díaz-Gimeno et al., 2011; Garrido-Gómez et al., 2013). This molecular tool can truly improve implantation rates (IRs) and pregnancy rates (PRs) for patients with recurrent implantation failures. With this tool, ART can become more precise and success rates can be improved.

#### Serum Autoantibodies and Female Infertility

The causes of infertility can be divided into three main categories: female causes (33–41%), male causes (25–39%), and mixed causes (9–39%). For females, the etiologies can be divided by organs into ovulation disorders [polycystic ovary syndrome (PCOS), premature ovarian insufficiency (POI), etc.], tubal infertility, endometriosis, and uterine and cervical disorders. However, the etiologies mentioned above cannot explain every couple’s problems. As a result, 8–20% of patients are labeled as “unexplained or idiopathic infertility” (Forti and Krausz, 1998; Leridon, 2007). For unexplained infertility couples, autoimmune disease might account for a portion of these cases. The risk of some identified autoimmune diseases, such as antiphospholipid syndrome (APS) and systemic lupus erythematosus (SLE), has been well studied. The correlation between serum autoantibodies and the prevalence of infertility and success rate of IVF or ICSI-IVF has been studied. Some experiments determined that autoantibodies such as APL, ANA, antitissue antibodies, thyroid autoimmunity, anti-ovarian antibodies, and other antibodies have a tendency of increasing the rate of infertility (Wilson et al., 1975; Hoek et al., 1997; Reimand et al., 2001; Shoenfeld et al., 2006; Abalovich et al., 2007), and APL, ANA, and anti-ovarian antibodies were noted as having a correlation with a low rate of implantation and pregnancy (Table 2; Kaider et al., 1999; Ying et al., 2012; Zhong et al., 2012). There is also a study showing that a statistically significant association exists between endometriosis and autoimmunity. These correlations provide new insights into unexplained infertility and offer clinicians more methods to determine the cause of infertility. Confirming the correlations that are already known prompts the infertility diagnosis into a more precise way. Still, the explicit mechanisms underlying autoimmunity and infertility are not fully understood. Autoimmunity tests and the studies mentioned above can only provide speculation rather than a specific explanation. More precise understanding is needed, and we hope someday that unexplained infertility will be explained (Deroux et al., 2017).

**TABLE 2:** Summary of the influence of antibodies on fertility.

**Antibodies**	**Female infertility**	**Art outcome**	**Treatment**
APL	Significantly associated with low levels of AMH (Vega et al., 2016).	Considered as a contraindication for IVF, increases the risk of thrombosis (Khizroeva et al., 2018).	Anticoagulant therapy from the first days of the hormonal protocol.
ANA	Increased prevalence of ovarian failure and infertility (Wilson et al., 1975; Taylor et al., 1989; Reimand et al., 2001).	Associated with higher abnormal fertilization and early miscarriage rates, a detrimental effect on IVF/ICSI outcome (Zhu et al., 2013).	Prednisone plus low-dose aspirin (P + A) adjuvant treatment.
Antitissue	Higher prevalence of anti-smooth muscle antibodies (Wilson et al., 1975; Reimand et al., 2001)		
Thyroid anti-immunity	Increases the prevalence of infertility, ovarian failure, and in particular, is related to endometriosis and PCOS.	Significantly increased risk of miscarriage (Poppe and Velkeniers, 2002).	Whether thyroid hormones should be given during pregnancy is still controversial (Poppe et al., 2006).
Anti-ovarian	Higher prevalence of ovarian failure and infertility (Ayesha et al., 2016).	Might be correlated to the prognosis of IVF	
Others	Higher prevalence of ASCA (Shoenfeld et al., 2006) and of CD-associated autoantibodies (Vanciková et al., 2002; Kumar et al., 2011; Machado et al., 2013; Tersigni et al., 2014).		

#### The Application of Precision Medicine in Gynecological Cancer

Gynecological cancer are a serious threat to women’s health. Patients with gynecological cancer accounted for approximately one-sixth of female tumor patients (Siegel et al., 2015). Among them, ovarian cancer, cervical cancer, and endometrial cancer are the most common gynecological malignant tumors, which have the characteristics of high incidence, high mortality, limited late efficacy, easy recurrence, and drug resistance. Gynecological cancer is a major threat to fertility for women of childbearing age. The diagnosis and treatment for tumor patients should be precise in time and space, and significant attention should be paid to gynecological cancer.

The conventional diagnosis of oncology is mostly based on pathology, and histological classification is nowadays gold standard for patient stratification. Histological type is an important predictor of survival and a determinant factor of surgery and adjuvant therapy. Endometrial carcinoma (EC) is the most common malignant tumor in female reproductive system, and is divided into two types in the pathogenetic point of view. Estrogen-related endometrioid carcinomas (EEC) and non-endometrioid carcinomas (NEEC). And WHO has an updated classification and nine subtypes are recognized (Kurman et al., 2014; Piulats et al., 2017). Different pathological subtypes correlates not only prognosis, but also molecular alternations. Like TP53 is mutated in >90% of serous carcinoma (SC), but this mutation is not exclusive for SC. This situation call on a classification combined histological types and molecular information. The Cancer Genome Atlas Research Network (TCGA) put forward a new classification of EC and divides EC into four groups. Group 1 refers to EEC with somatic inactivating mutations in POLE exonuclease and hypermutated (7%), prognosis is good. Groups 2 and 3 both showing similar progression-free survival rates. Group 2 included EEC with microsatellite instability (MSI), frequently with MLH-1 promoter hypermethylation and high mutation rates (28%). Group 3 included EEC with low copy number alterations (39%). Group 4 (Serous-like or copy-number high) (26%) showed low mutation rate, but frequent TP53 mutations, and worse prognosis, and was predominantly composed of most SC, and some EEC (Levine, 2013). This new classification might lead to better management of patients and more precise diagnosis of patients with EC. Some other types of cancer have also been explored into more precise classification, like ovarian cancer, uterine carcinosarcoma, and cervical cancer (Cancer Genome Atlas Research Network et al., 2017; Cherniack et al., 2017). Not only EC, the diagnosis of many other kinds of gynecologic cancer needs this optimization. Lynch syndrome (LS), a kind of hereditary cancer-prone syndrome, refers to high probability of several kinds of cancer like colorectal, gynecological, urinary tract, upper gastrointestinal, and other cancers. It may be caused by pathogenic variants of mismatch repair (*path_MMR*) genes, and usually four DNA *MMR* genes including *path*_*MSH2*, *path*_*MLH1*, *path_PMS2*, or *path_MSH6* are collectively refers to *path_MMR*. New evidence proved that genetic subgroups are at risk for different cancers in aging population and these cancers have very different prognoses. Like older *path_MSH2* carriers have higher incidence of urinary tract and prostate cancers, and older *path_MLH1* carriers have the highest incidence of upper gastrointestinal cancers. These information help clinicians estimate the risk of cancer and manage treatments better for patients with LS, and therapeutics can be designed on genetic factors. Treatment with LS can be improved, like ovarian cancers in LS were diagnosed before the menopause, and their prognosis was good, which brings questions into the necessity of prophylactic oophorectomy for female patients at the menopause with LS (Lynch et al., 2015; Møller et al., 2018; Dekker et al., 2019). Molecular information like integrating genomic, transcriptomic, and proteomic characterization can reveal differences of subtypes of cancer, especially those cannot be told and defined by histological classification, and better prediction of prognosis and more precise treatment can be given.

The treatment of gynecological cancer has become a pioneer in the application of precision medicine. Genetic screening and deciding whether to use targeted medicine have become routine for oncology clinicians. Ovarian cancer patients can benefit from olaparib (PARPi) if they are the *BRCA1/2* mutation type. The combination chemotherapy of Bevacizumab and non-platinum can significantly improve the response rate and progression-free survival time, and more importantly, overall survival was significantly prolonged (Tewari et al., 2017). The combination of targeted medicine and conventional radiotherapy and chemotherapy improves the efficacy of tumor treatment. However, precision medicine has not been widely used in gynecological oncology, and targeted medicine is not as efficient as we predicted because the pathogenesis of gynecological tumors is not fully understood. Bevacizumab, a type of vascular endothelial growth factor receptor (VEGFR) blocker, has been proven to be efficient in the inhibition of tumor metastasis, but no clinical statistics verify that it prolongs the overall survival rates of ovarian tumor patients. Some studies have identified some signaling pathways that are related to endometrial cancer pathogenesis, but effective targeted drugs are still lacking. In approximately 30–50% of cancer patients, it was possible to detect genes that could explain tumorigenesis and malignant transformation, but in only 3–13% of patients was it possible to find precise drugs, and only 30% of these patients experienced amelioration of disease even after finding the appropriate drugs. The final tally of those who benefited accounted for just 1.5% of all patients. Multiple factors can account for this limited success of targeted medicine, including limited access to targeted agents and inadequate tumor specimens and analysis. In addition, most molecular-targeted agents provide only partial inhibition of signaling pathways, and many are too toxic to be used in combination (Tannock and Hickman, 2016). Precision immunotherapy for cancer is developing and the immune checkpoint inhibitors (ICIs) had largely changed the landscape of cancer treatment, like non-small-cell lung cancer (NSCLC). However, the efficiency of immunotherapy is not satisfied, biomarkers for identifying the subset of patients who can clinically benefit from immunotherapy should be developed. Tumor mutation burden (TMB), PD-L1 expression, MSI, and other potential predictors can possibly give clinicians more precise identification of potential benefited patients and treat them with precision immunotherapy, and precision immunotherapy with gynecological cancer should be developed and explored (Johnson et al., 2016; McGranahan et al., 2016; Rizvi et al., 2018). Concerning all the deficiencies that exist in the application of targeted medicine, more research is needed to elaborate on the variability of the molecular characteristics of individual tumors and their relationship to the natural history and outcome of disease, and biomarkers for seeking precise treatment, and the combination of targeted drugs and other treatments should be explored.

Another concern in the treatment of gynecological tumors is preservation of female fertility. It is estimated that 15–20% of gynecological tumor patients are of reproductive age (Chen et al., 2015; Deroux et al., 2017). Current guidelines for most gynecologic tumors are inclined to genital dissection and lymph node dissection, which result in the loss of fertility. Additionally, radiotherapy and chemotherapy can seriously damage fertility. Reduced or lost fertility can seriously affect the quality of life of cancer survivors. The conception of fertility preservation has been presented, which refers to providing assistance to adults or children who are at risk of infertility through surgery, medicine, or assisted reproductive technology, protecting their reproductive endocrine functions, and hoping to obtain genetic offspring (Martinez, 2017). Fertility sparing surgery (FSS) has been applied in early gynecological malignant tumors. For early stage cervical cancer patients, if the lesion is in the I B1 or lower period without blood and lymph node invasion and the diameter is <2 cm, cervical resection to preserve fertility can be considered. The current clinical guidelines are still mainly based on imageological diagnosis. More specific clinical neoplasm staging is needed, and genetic information should be considered so that the surgical procedures can be more precise and unnecessary overtreatment can be avoided. To optimize neoplasm staging, genomic information can be combined with other treatments, such as ovarian transposition and oocyte cryopreservation (OC) (Noyes et al., 2011).

### Clinical Decisions

Evidence-based medicine enables clinicians to make clinical decisions based on clinical evidence, and precision medicine makes clinical decisions more individualized (Collins, 2017). The main task of EBM is the requirement for clinicians to weigh the effectiveness, safety, and costs of medical interventions. In reproductive medicine, safety and costs are frequently overlooked, although effectiveness is under long-lasting discussion. While EBM looks these three cornerstone of evidence in a broad way, clinical decisions made on these evidence can be more individual and precise (Martins et al., 2018). Clinicians can adjust the dosages of hCG based on patients’ remaining ovarian reserve and BMI, select specific fertilization techniques according to sperm features and clinical background, and monitor embryo development *in vitro* (Fang et al., 2018). In addition, gene profiles are a more precise guide to treatment. A genome-wide association study (GWAS) identified 11 genetic loci related to different phenotypes of PCOS. LHCGR is related to hyperandrogen, and FSHR is involved in the formation of PCOS ovulation disorder (Chen et al., 2010; Shi et al., 2012). The FSHB gene is related to hypertrichosis, and RAD50 is related to DNA repair (Diamanti-Kandarakis and Dunaif, 2012). Based on the mathematical model established by these risk genes, the individualized molecular quantitative detection of PCOS risk can be realized. Combined with clinical information, the prediction of PCOS genetic risk could be attempted, and preventive intervention measures such as controlling and improving diet structure could be implemented for high-risk patients (Tian et al., 2016).

Similar to PCOS, more individualized clinical decisions can be made while information about other diseases in reproductive medicine accumulates. Non-invasive diagnosis of fetal aneuploidy from cell-free DNA (cfDNA) in maternal plasma with the use of an Illumina HiSeq massively parallel sequencing platform had significantly lower false positive rates and higher positive predictive values for detection of trisomies 21 and 18 than standard screening. This technique offers clinicians more precise information of chromosomal variation and less chance of causing abortion. Clinicians can decide to perform further invasive examinations or treatment based on the result above (Bianchi et al., 2014). MiRNAs of placental origin are continually released in the maternal circulation throughout pregnancy, this might suggest that circulating miRNAs might serve as biomarkers for placental function during pregnancy. Clinicians might evaluate the function of placenta with the combination of different parameters like miRNA, DNA methylation, and traditional screening like Ultrasonography, and better prevention of adverse pregnancy outcome caused by placental dysfunction can be performed (Mouillet et al., 2015). Refined diagnosis will lead to specific and effective clinical treatment, higher safety, and less costs. These three main tasks for EBM still works for precision medicine, but with higher requirements. Clinicians can obtain more information and make decisions under individual situations rather than making a clinical decision for a group of patients, and that’s the superior part of precision medicine.

## Precision Medicine in the Future

Reproductive medicine has already accomplished a lot, but there is still a long road ahead to truly achieve precision medicine. There are difficulties in transforming the research results of genetics and biology into clinical application. Precision medicine might be able to fill this gap between basic research and clinical application.

### Mechanism Interpretation of Polygenic Diseases

Apart from the urgency of increasing the success rates and reducing the cost, reproductive medicine needs to be more elucidative. Complex diseases involve many variants, and identifying these determinants is a challenge for reproductive medicine. Mendelian diseases are controlled by a single locus, and the phenotype is easy to predict. Nevertheless, Mendelian diseases can only explain a small fraction of infertility causes, and a fairly large number of infertility causes cannot be explained by one single gene’s mutation. Many couples have received extensive diagnostic testing but never received a definitive diagnosis for their reproductive difficulties (Luke et al., 2012). The diagnosis of many causes of infertility is descriptive, such as recurrent pregnancy loss (RPL), decline in ovarian reserve (DOR), and POI. The mechanisms underlying these diseases are not clear. These uncertainties cause patients to have difficulties going through these time-intensive and emotionally taxing treatment cycles. We need to understand the mechanisms underlying symptoms and offer patients genetic-based tests and specific diagnoses and personalized treatments. We started noticing disease heterogeneity in oncology at the very beginning. Cancers are divided by organs and then subdivided by tumor characteristic molecules and genotypes, and targeted treatment is carried out based on this information, e.g., herceptin treatment is carried out in patients with HER2 overexpression. However, not enough attention is paid to disease heterogeneity in reproductive medicine’s diagnosis and treatment, as in oncology. Only 50% of the cases have a definitive diagnosis, and the definitive diagnosis for these 50% of cases is still heterogeneous. They might have disparate etiologies with distinct biological and molecular bases (Beim et al., 2013).

Multigene panel tests might reveal the mechanisms and lead us to a better understanding. For example, a multigene panel test can be used to assess ovarian function. Interventions can be offered on the basis of the multigene panel test results and clinical evidence (Noyes et al., 2011). Since genes at high risk and genes of lesser magnitude are all tested, multigene panel tests can reduce the likelihood of false-negative assessment and are cost-effective and time-efficient in oncology. As an ideal goal of reproductive medicine, identifying pathogenic genetic variants for patients with specific infertility phenotypes, such as ovarian dysgenesis or azoospermia, can be realized with focused gene panels (such as those offered by Centogene and Evolve Gene). Moreover, screening rare mutations that directly cause overt infertility, such as impaired oocyte maturation and fertilization defects, is also required. In 2017, Celmatix Inc. announced the first commercially available product, Fertilome, in the United States. Celmatix Lab obtained 49 specific single-nucleotide variants in 32 genes by masked analysis of targeted NGS, and Fertilome can offer services to patients with these data, which have been implicated in a variety of reproductive conditions, such as POI, RPL, PCOS, and endometriosis (Celmatix Inc., 2017). While the power of multigene panel testing depends on the amount of genetic information it provides, this level of gene information carries some inherent downsides. Identification of variants of unknown significance (VUS) can confuse clinicians and patients, for which there is inadequate evidence (e.g., from family or population studies, prior instances of the variant, *in vitro* or *in vivo* functional studies, or *in silico* predictions of function) to remark on its pathogenicity. We lack a thorough understanding of all the genes we test, and we might have difficulties explaining what the test results mean to patients. Some positive results of less relevant genes may cause anxiety for patients if clinicians cannot explain it properly. A close scrutiny of the literature in which risk alleles have been identified is needed to ensure clinical validity.

### Big Data for Precise Reproductive Medicine

Advancements in high-throughput omics technologies allow the generation of large volumes of biomedical data that can be usefully exploited for precision medicine. The big data and developing artificial algorithms provide the basis for novel insights into diseases that can be translated into personalized treatments (Krawetz, 2017).

Big data can offer help in randomized controlled trials (RCTs) and provide clinical evidence of high quality. In the field of reproductive medicine, big data can enable us to obtain more patient information, which is conducive to drug development, remote monitoring, and providing a lot of information for the exploration of the causes of infertility diseases. Genetic disease susceptibility genes have ethnic specificity, and big data can be used to find the specific susceptibility genes of the right population. Big data could help clinicians develop more specific guidelines for different situations and make more personalized determinations for patients.

As discussed above, reproductive medicine currently still has a low success rate owing to many patients’ explicit diagnoses. For patients without a defined diagnosis, intrauterine insemination (IUI) and IVF are still considered as treatment. Theoretically, these interventions should be given only if the expected success rates exceed the probabilities of spontaneous pregnancy. However, it is not easy for gynecologists to evaluate when objective criteria are lacking. Thus, prediction models are needed to help clinicians precisely estimate the success rate and make decisions that are in the best interest of the patient (Leushuis et al., 2009). Clinical studies have identified some predictors for the success rate of assisted reproduction technologies. Serum hormone levels including estradiol, FSH, and anti-Müllerian hormone, history of gestation, age and many other indicators are all strong predictors and are widely used in clinical decision and success rate predictions. These data can be integrated, and statistical and machine learning techniques are applied to derive an algorithm combining the clinical factors and yield a predicted likelihood of success for each patient, therefore making reproductive medicine more precise and individualized (Mayer-Schönberger and Ingelsson, 2018). Some clinical factor models based on logistic regression or Cox models are published but cannot be applied because of low currency. The algorithms need to be rectified, and iterative, adaptive, and learning models are needed (Leushuis et al., 2009). This algorithm can be repeatedly recalibrated by performing calculations on patients until its prediction coincides with real models (Beim et al., 2013).

**FIGURE 2:**
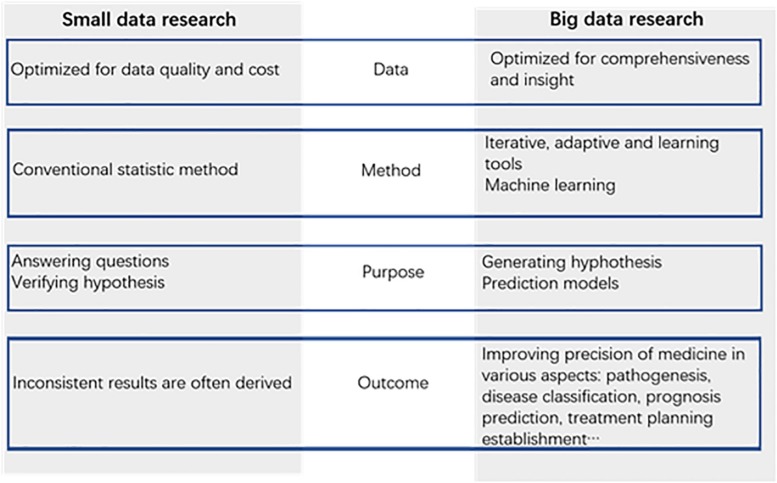
Comparison of conventional statistical methods and big data analysis.

Big data promise huge benefits for medical research. Apart from the prediction models, big data can offer us new insights in medical exploration. Unlike conventional analysis of data samples, big data can help us find some factors that we did not consider as causes for infertility. Data are captured more comprehensively relative to the phenomenon under study. This type of data collection reduces some bias and brings to the surface important trade-offs between data quantity and data quality. Data are analyzed by machine learning tools, such as neural networks, rather than conventional statistical methods. This learning system can capture insights implicit in data, but it rarely reveals causal connections, which remain as black boxes. The purpose of the analysis of data is no longer simply answering existing questions but hinting at novel ones and generating promising new hypotheses (Leushuis et al., 2009; Mayer-Schönberger and Ingelsson, 2018). Based on the characteristics of the big data mentioned above, we can note new factors related to the field of reproductive medicine and explore new directions for scientific research. Big data can narrow down the range of factors that need to be studied and help make medicine research more precise (Figure 2).

### Drug Screening With Precision Medicine

Targeted molecular medicine has been widely studied in oncology, and many targeted drugs have already been applied clinically. Many drugs have been developed; however, not many have clinical benefits. In addition, drug resistance is an issue that cannot be ignored.

Since the first biotechnology-derived pharmaceutical recombinant human insulin was introduced and licensed for human use in 1982, huge progress has been made in molecular biology, functional genomics, and proteomics, and the number of large molecule biopharmaceuticals for human use has rapidly increased. Unlike conventional small-molecule drugs that are chemically synthesized, biopharmaceuticals cannot be tested in standard processes. Biopharmaceuticals are derived from living sources, such as humans, animals, plants, and microorganisms, using manufacturing processes that are often complex. Most biopharmaceuticals are large molecules or heterogeneic mixtures of large molecules that are not easily characterized, often display species specificity and pleiotropism in their pharmacologic effects, are immunogenic, and tend to be heat-sensitive and susceptible to microbial contamination (Brennan et al., 2004).

Identification of effective combination therapies is critical to address the emergence of drug-resistant cancers, and many new screening techniques have been developed. The CRISPR-based double knockout (CDKO) system provides new possibilities for cancer medicine screening. This screening method combines genomic information so that it can proceed in a precise way (Han et al., 2017). For reproductive medicine, apart from the targeted drugs for gynecological cancer, many causes of infertility, such as PCOS and endometriosis, are associated with molecular mechanisms, and some drugs have been developed addressing these mechanisms. Similar to anastrozole and letrozole, third-generation aromatase inhibitors, which selectively inhibit the action of aromatase, have been investigated for the treatment of endometriosis as monotherapy or in combination with other hormonally active drugs (Ferrero et al., 2018). Atosiban, a mixed vasopressin V1a and oxytocin receptor antagonist, has been registered for the treatment of imminent premature birth with minimal side effects. Now atosiban and its advanced version barusiban can reduce uterine contractions, and increase the clinical PR per cycle and IR per transfer (Blockeel et al., 2009; Visnova et al., 2009; Moraloglu et al., 2010). Besides, efforts on targeted drug delivery system also bring new angle of precision medicine. Mesoporous silica, a kind of targeted nanovectors for biological delivery facilitate the precise transport of large amounts of any type of compound to specific destinations, followed by rapid internalization via physiological uptake mechanisms and the ultimate targeting of specific intracellular pathways (Barkalina et al., 2014). Other drugs are being developed for different molecular mechanisms, and gene editing technologies can also be used to screen drugs for effectiveness and personal fitness. However, targeted medicine are not well developed in the area of reproductive medicine like oncology does, even though molecular mechanisms and genomic variations of reproductive diseases are widely studied and explained. We hope cancer drug screening system can be applied in the field of reproductive medicine and search for more targeted drugs aims at genetic variations and signaling pathways.

The research on stem cells brings us a promising future wherein stem cell therapy can be possible. For some patients seeking the help of ART, there is the problem of them having no functional gametes. Donor gametes can solve the problem but cannot be accepted by every couple, especially those people who want their genetically own offspring. The emergence of induced pluripotent stem cells (iPSCs) (Takahashi and Yamanaka, 2006; Takahashi et al., 2007) brings new solutions to this problem. Gametes induced by autologous cells offer possibilities for people without functional gametes. However, the safety is uncertain, and it cannot be tested in conventional procedures because iPSCs induced from one person’s somatic cells can only be tested on himself/herself (Vassena et al., 2015). A case report about a patient treated with retinal pigment epithelium derived from human embryonic stem cells indicates that stem cell therapy is on the brink of being applied in other medical specialties (Schwartz et al., 2012). There are no stem cell-based therapies available to the larger public or outside of clinical trials directed at ameliorating or solving reproductive medicine issues yet. However, we believe that this day will come.

## Conclusion

Starting from oncology, precision medicine has already permeated into various fields of medicine. For reproductive medicine, precision has always been a criterion in every procedure, including etiology-oriented examination, specific diagnosis, identifying healthy embryos, WOI, and accurate implantation. Combined with genetic information and a large volume of biomedical data, an unknown territory of reproductive medicine will be explored, and the mechanisms underlying the causes of infertility that we do not yet know will be elucidated. The application of precision medicine has become a guideline for the development of medicine, especially for reproductive medicine.

In many other specialties, more and more available clinical and genetic biomarkers have helped physicians make overall disease prediction and have informed clinical decision making. Chronic diseases such as cancer, cardiovascular disease, and type-2 diabetes place a heavy burden on society, and the health care model is gradually evolving into a proactive system. A 4P health care model (Predictive, Preventive, Personalized and Participatory) has been put forward and has been increasingly popularized in the care of chronic disease. The outcome for a breast cancer patient can be predicted by the expression of HER2 and ER; likewise, can the risk of infertility and success rate of ART be predicted? Can this 4P model be applied to reproductive medicine? Can the application of the 4P health care model make reproductive medicine more precise? We believe the answers are affirmative. Regarding prediction, avoiding the accumulation of stressors and identifying early symptoms are primary objectives. For reproductive medicine, current biomarkers such as hormone levels and ultrasound examination are still at the core of predicting disease. The next generation of biomarkers combined with big data technology will increase the precision of identifying dysfunctions. Information on the genome, epigenetic groups, transcriptome, proteome, metabolome, and molecular diagnosis can be integrated, and based on this information, screening and identification platforms can be established. For prevention, the essence is avoiding the causes of infertility. Among all the causes of infertility, some are related to an unhealthy lifestyle. Strengthening education on female reproductive health will be a strong prevention for infertility. With our deepening understanding of different causes of infertility, more personalized diagnoses can be made, and more precise treatment can be given, and in this way, the success rate of ART can be improved. Meanwhile, it is important to strengthen the psychological treatment and education of patients to overcome the emotional barriers and prevent dropout caused by a lack of awareness. Involving the individual in personalized treatment and preventive interventions and improved data collection through self-tracking will be important factors. To achieve this goal, we have to gain a much better understanding of an individual’s “health literacy” (Sagner et al., 2017). With the continued promotion of precision medicine, reproductive medicine will reach new heights and bring hope for more patients who are suffering from infertility.

## Author Contributions

P-YZ drafted the manuscript. YY conceived the idea and revised the manuscript.

## Conflict of Interest

The authors declare that the research was conducted in the absence of any commercial or financial relationships that could be construed as a potential conflict of interest.
